# Radio Frequency Treatment of Food: A Review on Pasteurization and Disinfestation

**DOI:** 10.3390/foods12163057

**Published:** 2023-08-15

**Authors:** Daniela Bermudez-Aguirre, Brendan A. Niemira

**Affiliations:** Food Safety and Intervention Technologies, ERRC, ARS, USDA, 600 E Mermaid Lane, Wyndmoor, PA 19038, USA; brendan.niemira@usda.gov

**Keywords:** radio frequency, thermal processing, volumetric heating, pasteurization, disinfestation, dielectric properties

## Abstract

Radio frequency (RF) is a novel technology with several food processing and preservation applications. It is based on the volumetric heating generated from the product’s dielectric properties. The dielectric properties of each material are unique and a function of several factors (i.e., temperature, moisture content). This review presents a list of dielectric properties of several foods and describes the use of RF as an innovative technology for the food industry. This paper includes several examples of pasteurization, fungi inactivation, and disinfestation in selected food products. The aim of this review is to present the potential applications of RF in pasteurization and disinfestation and research needs that should be addressed. RF has been successfully applied in the inactivation of pathogens such as *Salmonella* spp., *Listeria monocytogenes*, and *Escherichia coli* in low- and high-moisture food. The disinfestation of crops is possible using RF because of selective heating. This process inactivates the insects first because of the different dielectric properties between the pests and the food. The products’ final quality can be considerably better than conventional thermal processes. The processing time is reduced compared to traditional heating, and thermal damage to the food is minimized. The main drawback of the technology is the lack of uniform heating, mainly when the product is surrounded by a packaging material with different dielectric properties from the food.

## 1. Introduction

For several decades, conventional thermal processing has been the preferred technology to pasteurize and sterilize food. It is based on applying heat from an external source, such as water, steam, or air. However, the heat can degrade the nutritional quality and affect the product’s sensory characteristics. Regarding energy and cost, it is highly desirable to reduce the processing time. From the microbiological point of view, pasteurization and sterilization are needed to provide a safe product. Both thermal technologies ensure the microbial quality of the product, but both processes adversely affect the food’s bioactive compounds and sensory attributes. 

On the other hand, foodborne outbreaks become more common every year. Primary pathogens such as *Salmonella* spp., *Escherichia coli* O157:H7, and *Listeria monocytogenes* are frequently associated with these outbreaks. The Centers for Disease Control and Prevention [1] reports a list of outbreaks involving a diverse number of food products yearly. Some microorganisms, such as *Salmonella* spp., have been resistant to different food environments, such as refrigeration temperature, low moisture content, and low- and high-acid foods. It is possible to find this microorganism in foods, such as flour, baby formula, produce, peanut butter, spices, juices, eggs and poultry products, and fish, and the list is endless. Many pathogens have built resistance to conventional technologies, and they have become adaptable to stress factors such as extreme pH, temperature, and water activity conditions, to mention a few [2,3]. Then, any food could be a good vehicle for pathogens if safety precautions are not taken during food processing. 

Emerging technologies surged as an option to improve food quality but also to ensure the microbial safety of the product. Most of the novel technologies offer faster microbial inactivation preserving the sensory and nutritional attributes of the food. In the area of emerging thermal processing technologies, radio frequency (RF), microwave, and ohmic heating are just a few examples. Radio frequency (RF) is energy that belongs to the electromagnetic spectrum and, as a novel thermal technology, uses the intrinsic properties of food for heating. Unlike conventional thermal processing, RF generates heat inside the product, and because of that, the volumetric heating is quick, and the required time to inactivate microorganisms is considerably less. This short processing time allows for maintaining the nutritional and sensory properties of the product almost without change. It also provides a more efficient technology from the energy and cost points of view. RF has also been tested for other applications in food processing, such as disinfestation of products, processing aid during drying, thawing and baking, cooking, tempering, and roasting [4,5]. This manuscript reviews this technology as an option for food pasteurization and disinfestation, highlighting the advantages of RF but also those research gaps that need to be researched in the next coming years. 

## 2. Basic Principles

RF is a novel thermal technology based on the volumetric heating of the product. This technology uses the dielectric and thermal properties of the food to generate heat from inside the product. The range of frequencies in the electromagnetic spectrum for radio frequency extends from 1 to 300 MHz. However, there are specific frequencies for Industrial, Scientific, and Medical (ISM) uses. The Federal Communications Commission (FCC) allowed for the use of radio frequencies for heating at 13.56 MHz, 27.12 MHz, and 40.68 MHz [4,5]. These restrictions in using specific frequencies avoid interference with other radio frequency areas, such as cellphone telecommunications. 

**Figure 1:**
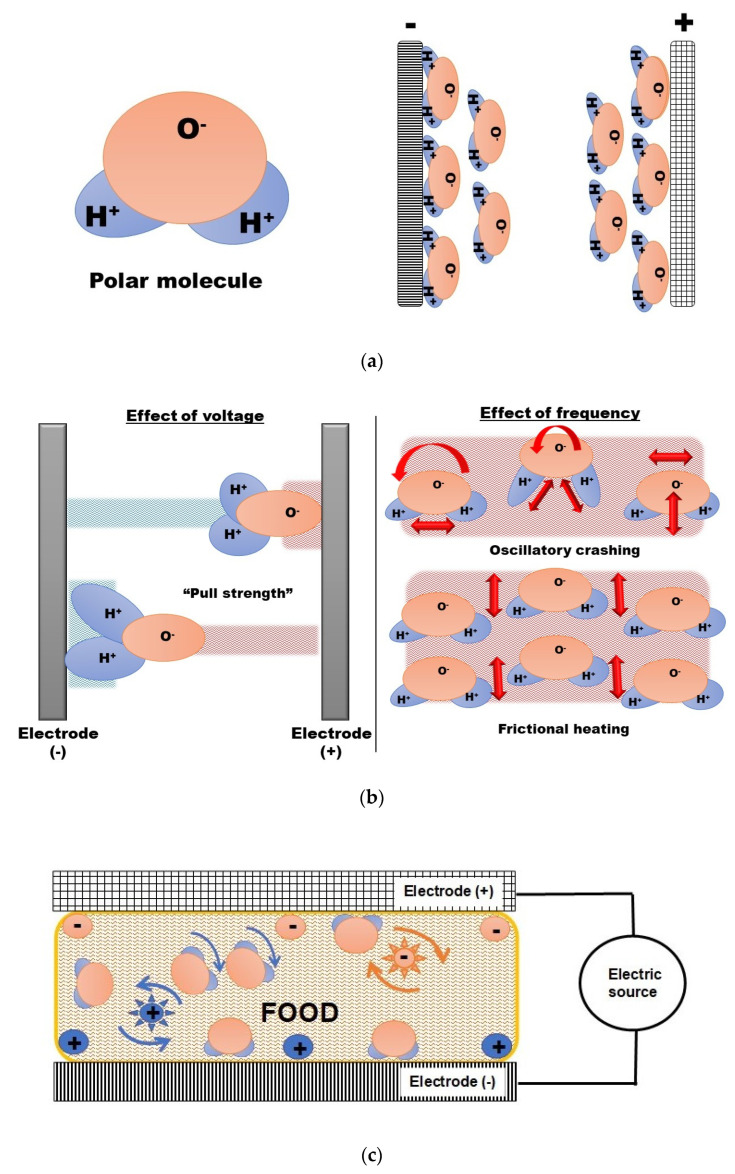
Schematic representation of polar molecules and radio frequency heating. (**a**) A polar molecule (i.e., water) aligns against electrodes, (**b**) the effect of the electric field has a “pulling strength” on the polar molecules; the effect of frequency produces oscillatory crashing and frictional heating, (**c**) dielectric heating in foods is based on the collision of ions aligning to the electric field and the friction between molecules because of the dipole rotation.

When a material is in contact with an alternating electric field, the thermal and electrical properties of the product will react to this interaction. The ions in the material will attempt to migrate to the opposite pole of the electric field; in other words, positive ions will move close to the negative pole of the electromagnetic field and vice versa [4,5]. This is called ionic migration or conduction (Figure 1a). Furthermore, the rotation of dipole molecules, such as water, aligning to the electromagnetic field will generate heat because of friction effects. In an oscillating magnetic field, these movements are reversed thousands of times every second. The heating effect of radio frequency is the consequence of the collision of ions inside the food and the friction between dipole molecules (Figure 1b, c). These physical phenomena are in some way similar to microwave heating and are illustrated in Figure 1.

### 2.1. Dielectric Properties

The dielectric properties of food are critical during volumetric heating because they will determine how uniformly and how quickly a product will heat when exposed to an oscillating electric field [6]. In other words, the dielectric properties of food quantify how the energy from the radio frequency wave is reflected, stored, or utilized [7]. 

The dielectric properties are related to the relative permittivity (*ε*), which determines the ability of a material to interact with an electromagnetic field, as shown in Equation (1) as follows:

(1) $$\varepsilon =\varepsilon^{\prime }-{j\varepsilon }^{\prime \prime }$$

where *ε*′ is the material’s dielectric constant, and *ε*″ is the dielectric loss factor. 

The dielectric constant *ε*′ is a measurement of the material or food to absorb, transmit, or reflect electromagnetic energy. The loss factor *ε*″ provides information about the ability of a material to dissipate energy during heating. It gives a measurement of the energy lost from the electric field. A low value of *ε*″ means the material absorbs less energy and does not heat appropriately with RF [4]. The penetration depth (*d_p_*) is the distance below the surface at which the power is reduced to 1/*e* (*e* = 2.718) or 36.9% of its original value [8]. It can be calculated as follows with Equation (2):

(2) $$d_{P}=\frac{c}{2\pi f\sqrt{2\varepsilon^{\prime }\left[\sqrt{1+{\left(\frac{\varepsilon^{\prime \prime }}{\varepsilon^{\prime }}\right)}^{2}}-1\right]}}$$

where *c* is the speed of light (3 × 10^8^ m/s), and *f* is the working frequency of the RF equipment [7]. RF shows a better penetration depth compared to microwaves. This characteristic makes RF ideal for processing large unpackaged products [9]. All these properties are a function of temperature, frequency, density, moisture content, and food composition. Other important properties of food when working with RF are the electrical conductivity (S/m), the thermal conductivity (W/m K), and the specific heat capacity (J/kg K). 

The dielectric properties of food can be adjusted to specific values to improve the heating of the product. These modifications are made when the product’s composition is changed (i.e., adding salt, modifying the water content, or changing the water state). 

There are several methods to quantify the dielectric properties of food, including the following:

a. Open-ended coaxial probe (OCP). This method allows for the quantification of the permittivity in semisolids and liquids. The sample preparation is easy, and the results have high accuracy. The probe of the equipment works by flashing signals; in solids, the probe touches a flat surface of the material, and in liquids, the probe plunges inside the product. The main drawback is the presence of air gaps that can provide erroneous measurements.
b. Transmission line method (TLM). This methodology has high accuracy and sensitivity for solids and liquids, though the main limitations are the restricted range of frequencies (<100 MHz) and the time-consuming sample preparation. The dielectric properties are quantified via the phase and amplitude of a microwave signal reflected from a material sample placed by the end of a transmission line.
c. Resonant cavity method. This method is suitable for high-temperature solid materials and is the most accurate. The sample is placed in the middle of a waveguide, and changes in frequency are recorded. This method is quick, and sample preparation is easy (non-destructive). However, the analysis of data can become complex.
d. Parallel plates. The sample is placed between two electrodes, and an alternate current is applied. The sample needs to be added as a flat sheet. It is an inexpensive and highly accurate method but with limited frequency (20 Hz–1 GHz).
e. Free space. In this method, the solid sample is placed between two antennas to apply energy together with a vector network analyzer. Samples are analyzed in the microwave range, a non-contact, non-destructive technique.

The easiest method is the first one. It does not damage the sample, provides measurements in broadband, and does not require specific containers. The other methods are more accurate but are time-consuming, and the measurements are limited to particular frequencies [10]. 

#### 2.1.1. Effect of Moisture Content

Water is found in food as free water or bound water. Free water is available for microbial growth; bound water is part of the food components attached to proteins, carbohydrates, and other molecules. The water molecule has a high polarity, and when water molecules are exposed to an electric field, the molecules rotate, aligning to the electric field, generating heat (see Figure 1). The free water in food has similar dielectric properties to liquid water; bound water has dielectric properties like ice. As the moisture content of the product increases, the dielectric properties increase too. However, the dielectric properties depend on the temperature of low-moisture food. If the temperature increases, the dielectric properties increase [10]. Table 1 shows a clear example of the effect of moisture content on the dielectric properties. Comparing two products, Red Delicious apples (87% moisture content) to dried apricots (24.6% moisture content), at 20 °C, the dielectric constant is about two times higher for apples, regardless of the frequency.

Another example is presented in Table 2 for milk. The dielectric constant for raw milk (88.20% moisture content) is 90.4 (27.12 MHz, 20 °C). The dielectric constant for whole milk powder (1.8% moisture content) is 1.51 at the same frequency and temperature. Table 2 presents a compilation of dielectric properties for animal food products and the effect of the moisture content. Meanwhile, Table 3 shows several examples of low-moisture food like flour, spices, and nuts; these products have a low dielectric constant. 

#### 2.1.2. Effect of Temperature

The effect of temperature on the dielectric properties is a complex relationship. In general, when the temperature increases, the dielectric constant also increases. However, the changes are a function of the frequency of the loss factor. For instance, at low frequencies, the loss factor will increase as the temperature rises, and it is because of ionic conductance. As previously defined in Section 2.1, the loss factor provides information about how easily the material can dissipate energy during heating. At high frequencies, the loss factor decreases as the temperature rises because of free-water dispersion [10]. Table 2 presents the effect of temperature on the dielectric constant (*ε*′) of chicken breast. At 27.12 MHz, *ε*′ is 91.64 when the temperature is 20 °C, but *ε*′ is increased to 109.18 when the temperature reaches 60 °C. For chickpea flour, the dielectric constant reported at 20 °C is 2.99, and this constant at 90 °C is 11.20, with both values at the same moisture content (7.9%) and same frequency (27.12 MHz), as presented in Table 3.

#### 2.1.3. Effect of Food Composition

Some food components, such as salt or fat content, can affect the dielectric properties of the product. For example, the presence of salt can change the dielectric constant of the food because of ionic conduction. The material’s loss factor (*ε*″) will increase in a product with high salt content as the temperature and frequency increase [4]. This fact can be observed in Table 2 for fish and seafood. The example of salmon caviar, containing 2.3% salt, has a loss factor (*ε*″) of 1349.4 at 20 °C. Comparing the same product at the same temperature and frequency but with low salt content (0.8%), the loss factor decreased to 470.8. Another example is presented in Table 3 with pecan kernels with no salt (*ε*″ 3.35), light salt (*ε*″ 8.58), medium salt (*ε*″ 14.96), and heavy salt (*ε*″ 24.26).

The ash content of food can also impact the dielectric properties. The minerals present in foods and those non-organic components represent the ash content. Ashes can bind some water ions reducing the functionality of these volumetric heaters. Products with low ash content, such as fruits and vegetables, do not significantly affect the dielectric properties. Products with higher ash content are expected to have a lower dielectric constant and higher loss factor [10]. 

The effect of fat content in food has also been studied during RF heating. One study by Farag et al. [47] using beef (lean, fatty, 50:50 mixture) showed that fat does not allow for the proper heating of the product. The three products showed an initial temperature increase when the radio frequency heating started, but the fatty product showed a quick decrease in the temperature. A specific example is presented in Table 2 for pork. The dielectric constant of lean pork is 69.6 (27.12 MHz) and decreases to 12.5 when the pork has high-fat content. 

#### 2.1.4. Other Effects

In the case of heterogeneous mixtures, such as granular or particulate materials, the product’s bulk density will impact the food’s dielectric properties. This is important when processing grains, seeds, and spices [6,10]. During processing grains or seeds, there are void spaces in the bulk material. These spaces are filled with air. Then, the dielectric properties of air must be considered during the evaluation. 

Studies of the dielectric properties of diverse food and materials have been widely reported. Since the knowledge of these properties represents the foundation for developing radio frequency treatments and equipment, quantifying these properties has been extensively reported in the literature. Table 1, Table 2 and Table 3 present a compilation of some of the electrical properties reported for fruits and vegetables, animal products, and miscellaneous food. The values in the tables belong to the most common frequency used for food applications (13.56, 27.12, and 40.68 MHz). Also, the values are presented at different temperatures and, in some cases, at additional moisture content. Specific values at a particular temperature or processing conditions are available in some literature reports. 

## 3. Equipment 

**Figure 2:**
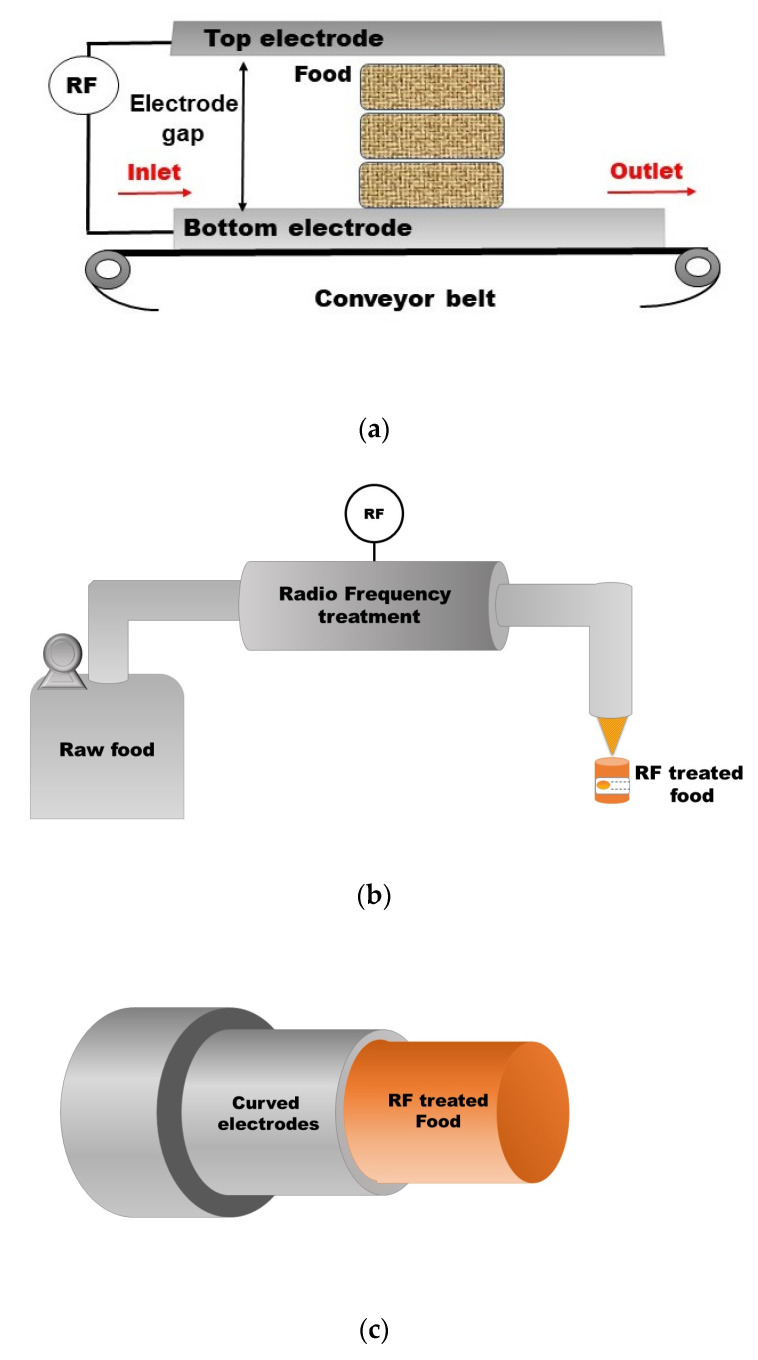
Schematic representation of radio frequency equipment (**a**) Semi-continuous mode for solid food in which the system uses a parallel plate array of electrodes; (**b**) continuous mode for liquid pumpable food; (**c**) curved electrodes inside of a tubular RF system.

The most basic RF equipment consists of a couple of electrodes connected to a radio frequency source. The food is placed between two electrodes to apply the electric field. The gap between the electrodes can be modified to adjust the intensity of the treatment. Figure 2a is a schematic representation of a simple RF semi-continuous equipment. This piece consists of two parallel electrodes connected to the alternating radio frequency energy source. The chamber is loaded with food on the left and the processed product leaves on the right. The system can operate semi-continuously if a conveyor belt is added to move the product. Another electrode configuration is the stray field (fringe field), which consists of a series of electrodes with specific shapes, such as bars, rods, or narrow plates. Depending on the thickness of the food, different electrode arrangements can be used for processing. For example, the staggered through field electrodes are preferred for regular products with a thickness of about 6 mm, and these are the best choice for thawing. The stray-field electrodes are selected for products like sheets with thicknesses less than 1 mm. Meanwhile, parallel plate electrodes are the best option for bulk, thicker and large products [48]. Some RF systems are equipped with an auxiliary hot air system. This air will help maintain the product’s temperature and increase the process’s lethality [49]. 

For liquid and pumpable products, the RF systems consist of tubular sections. The product is pumped through a tube with curved electrodes attached to the system. The liquid receives the treatment, and the heating results from the dielectric properties. This equipment has been tested for some salt solutions and milk [48]. A schematic representation of a liquid RF system is presented in Figure 2b,c.

**Figure 3:**
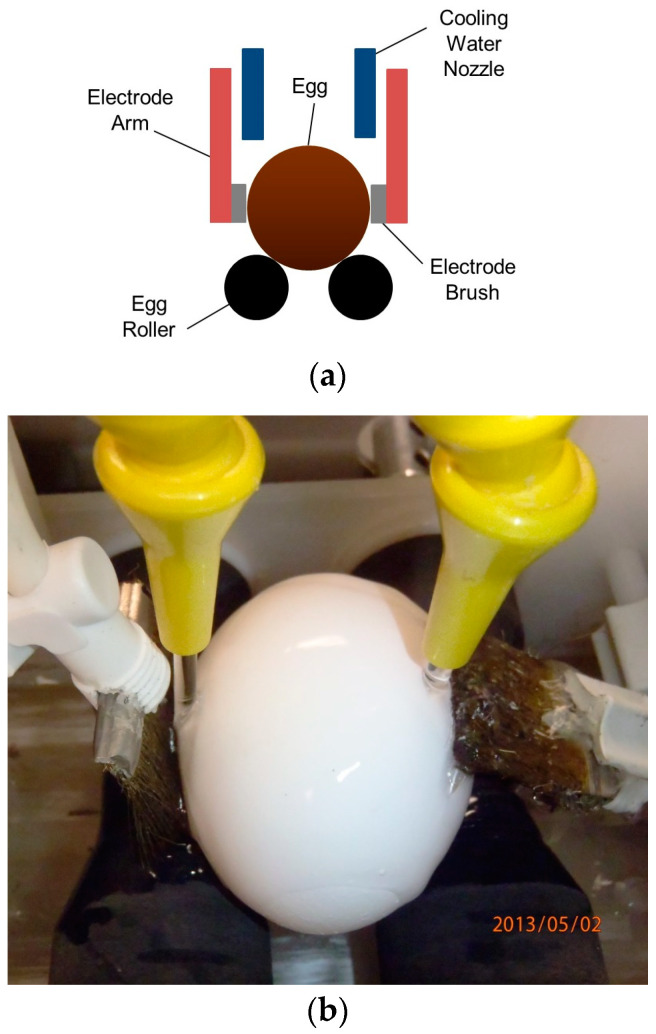
RF equipment to pasteurize in-shell eggs with a parallel plate array of electrodes, (**a**) Schematic representation [52]; (**b**) actual view of the system (Photo credit: USDA ARS ERRC).

In Figure 3, there is a representation of an RF system explicitly developed to pasteurize in-shell eggs. In this system, the eggs are rotated during the processing to allow for more uniform heating and reduce the problem of hotspots. The cooling water nozzle is used to spray cold water during the process and reduce the shell’s temperature to protect the albumen from thermal damage. The active and ground electrode are conductive brushes to transfer the energy into the product. This system has been successfully adapted for use in combination with hot water immersion (HWI), hot water spraying (HWS), or hot air (HA) to reduce processing time [50,51,52]. A schematic diagram of the egg and the electrodes is shown in Figure 3a. The former electrodes of the existing RF system are shown in Figure 3b.

Some critical factors during RF processing are the size, shape, orientation, homogeneity, and food location inside the equipment [49]. Zuo et al. [53] studied the effect of different sizes and densities of walnuts during RF treatments (27.12 MHz, 6 kW). Samples were placed in containers, and the thickness was measured. Whole and half walnut kernels were used, as well as cracking pieces. The heating rate was higher as the sample was thicker, but the heating uniformity was worse. However, as the heating rate decreased because the electrode gap was bigger, the uniformity was improved. 

There is a mathematical equation to express the uniformity of RF heating, and it is called the RF heating uniformity index (*λ*), and it is described as follows in Equation (3):

(3) $$\lambda =\frac{\sqrt{\sigma^{2}-\sigma_{0}^{2}}}{\mu -\mu_{0}}$$

where *σ*_0_ and *σ* are the initial and final standard deviation of the food temperature before and after processing, and m and m_0_ are the final and initial temperature of the food. The lower the value *λ*, the better the heating uniformity [53,54]. The heating uniformity index (*λ)* increased as the kernel size in the previous study related to walnut kernels increased, but *λ* was decreased when the density of the kernels was increased. It was observed that mixing kernels (whole, halves, and cracking pieces) improved *λ* [53]. 

One of the main challenges today is the heating uniformity of the product when scaling up the technology. Some factors that affect the process’s uniformity are the product’s size and shape and the position between the electrodes. Several attempts have been made to improve the uniformity of the process, such as the rotation of the product or using a conveyor system to move the product inside the equipment, water immersion of the product, stirring/mixing, hot water preheating, hot air assistance, changes in the composition of the product, changes in electrode shapes, and adding some dielectric material to the product, among others [9,14,49,54,55,56,57,58]. Hao et al. [54] tested using a rotation device with sample mixing during RF heating of some granular products. Several variables were evaluated, such as the rotation speed, the product’s moisture content, and the product size. The products included mung bean, coix seed, peanut kernel, and almond kernel. The authors concluded that the lowest value *λ* was for mung beans because of their small size and moisture content (8.15%). 

It is also clear today that the heating uniformity of the product is affected by the dielectric properties and the density of the surrounding medium, often the packaging material [56]. In packaged food using rectangular containers, the main problem during RF is edge overheating. This is because of the higher energy absorption by the edges and corners of the packaged product. This different energy absorption is due to the differences in dielectric properties between the food and the packaging material [56]. Furthermore, some studies have indicated that the area of better heating when a product is placed between two electrodes is the central layer of the product. Also, when comparing the product’s central point and edges, the latter seems to be better heated with RF [58]. Ideally, the packaging material should have a low density or a close value to the product treated with RF. If dielectric properties are similar between the food and the packaging material, the nonuniform heating will be reduced drastically, as the computer simulation experiments show [59].

RF is not only a quick and accessible technology to apply to food products. Besides the remarkable improvement in sensory and nutritional attributes of the treated product compared to conventional thermal processing, there are significant savings in energy and costs. RF can be considered a green, non-contact, waterless, and energy-efficient technology [9,60]. Some energy calculations were conducted in a detailed study to scale up an RF system. For a continuous system used for the disinfestation of walnuts, the energy or heating efficiency had an average value of 79.5%. This value was greatly improved by mixing the product, moving the product in a conveyor belt, and adding hot air to the processing [49]. 

## 4. Food Pasteurization

Pasteurization is a thermal process applied to food to inactivate pathogens, spoilage microorganisms, and enzymes, extending the product’s shelf life. Food is usually heated at temperatures below 100 °C for several seconds or minutes [61]. Some inactivated pathogens via pasteurization are *Salmonella*, *L. monocytogenes*, *E. coli*, *Staphylococcus aureus,* and *Cronobacter sakazakii.* Pasteurization aims to reduce at least 5-log or 99.999% of the pathogen of concern in the product. However, to achieve this log-reduction, the processing time is long enough to degrade some nutrients such as vitamins and negatively affect sensory attributes like color or texture. Then, the need for an alternative for pasteurization using shorter processing times but ensuring the microbial quality of food brought radio frequency as an option. RF can achieve pasteurization standards in food products [57,61], and microbial inactivation is often higher than in conventional pasteurization [48]. 

The microbial inactivation because of RF is attributed to thermal effects on the cells [8]. The effects of the thermal treatment on microbial cells are mainly in the ribosomal DNA and the exhaustion of Mg^+^, which are vital for metabolic processes. Protein coagulation has also been reported as one of the lethal effects of heat [62]. The thermal inactivation of the microorganisms occurs when the cell generates heat faster than the surrounding media. It is affected by the composition of the media but also by the organism itself. It is believed that there is a mechanical disruption of the cells when subjected to RF because of the continuous, rapid oscillation when re-aligning to the oscillating electric field. This constant realignment breaks the cellular membranes when the elastic limit of the cell is exceeded, leading to cellular death [63]. However, today, there is still controversy about the main inactivation factor during RF. Perhaps, it is likely correct to believe that the inactivation of microorganisms is due to a synergistic effect between thermal and mechanical effects of the radio frequency energy in the cell membrane and intercellular structures. 

This novel thermal technology has been tested with successful results in the pasteurization of milk, meat, spices, nuts, flour, and eggs [9]. RF pasteurization can be an attractive option for those products with low moisture content, such as nuts. Conventional methods require very long processing times to ensure heat transfer in the product. Also, pathogens are more resistant to inactivation in low-moisture products [48]. RF offers the advantage of the product’s internal heating, reducing the processing time considerably. A comprehensive list of pasteurized products using RF is presented in Table 4. These references belong to those experiments that showed at least a 5-log reduction of the pathogen in the food product. However, several references report pathogen inactivation at a lower degree. Some of these studies present possible improvements in the equipment and/or the processing conditions to enhance microbial inactivation. 

Most of the current research shows hurdle technology as an option, combining several factors with RF to increase microbial death [64,65]. A few examples are presented in Table 4, for example, sesame and flaxseed seeds during the inactivation of S. Montevideo and S. Typhimurium. Xu et al. [64] treated the seeds with plant essential oils and RF to increase cell inactivation. These authors tested cinnamon vapor oil and oregano oil vapor. The best result was obtained with RF heating at 80 and 85 °C for 5 min and 0.83 μL/mL of cinnamon vapor oil for three days. This combination achieved more than a 5-log reduction of *Salmonella* cells.

RF can potentially be used in products involved in foodborne outbreaks reported recently. For example, a recent foodborne outbreak in 2022 in infant formula involved *C. sakazakii* or the presence of *E. coli* in cake mixes in the 2021 outbreak [1]. Several reports have also mentioned the presence of *Salmonella* spp. in flour. These products and microorganisms have been studied successfully, as shown in Table 4. RF can also be a valuable tool to treat spices that often contain high microbial loads if other interventions are unavailable or not practical. These high microbial concentrations in spices can limit the development of new products in the food industry and promote cross-contamination during the food production chain. 

**Table 4:** Examples of pasteurization and fungi inactivation in food products using radio frequency.

Pasteurization *				
Product	Microorganism	Processing Conditions	Log Reduction	Reference
Corn flour	*Salmonella enterica* Enteritidis PT30	27.12 MHz, 6 kW, 85 °C, 10 min, followed by −20 °C 48 h	6.6	[66]
Wheat flour	*Salmonella* Enteritidis PT30	27.12 MHz, 6 kW, 30 min	>5	[67]
Liquid whole egg (LWE) and liquid egg yolk (LEY)	*Salmonella Enteritidis*	27.12 MHz, 12 kW, 180–285 s	5.6 (LWE) 5.3 (LEY)	[68]
Cumin seeds	*Salmonella enterica*	27.12 MHz, 6 kW, 90–106 s	>5.8	[69]
Sesame seeds	*Salmonella* Montevideo and *Salmonella* Typhimurium	27.12 MHz, 6 kW, 80 °C, 5 min, plus 0.83 μL/mL Cinnamon Vapor Oil	>5	[64]
Flaxseed seeds	*Salmonella* Montevideo and *Salmonella* Typhimurium	27.12 MHz, 6 kW, 85 °C, 5 min, plus 0.83 μL/mL Cinnamon Vapor Oil	>5	[64]
Ground black pepper	*Salmonella* spp.	27.12 MHz, 6 kW, 130 s	5.98	[70]
Paprika	*Salmonella* spp.	27.12 MHz, 6 kW, 80 °C, 5 min	>6	[71]
Shell egg	*Salmonella* Typhimurium	27.12 MHz, 1 kW, 56.7 °C, 21 min	>6.1	[52]
Black pepper kernels	*Salmonella* Typhimurium ATCC 14028	27.12 MHz, 12 kW, 100 °C, 8 min	>6	[72]
Basil leaves	*Salmonella* spp. and *Enterobacter faecium*	27.12 MHz, 6 W, 65 s	>6.5	[73]
Buckwheat kernels	*Salmonella* Typhimurium, *Escherichia coli* ATCC 25922, *Cronobacter sakazakii*	27.12 MHz, 6 kW, 85 °C, 5 min	≈5	[74]
Cocoa powder	*Enterobacter faecium* NRRL B-2354	27.12 MHz, 6 kW, 75 °C, 48 min	5.5	[75]
Almonds (in-shell)	*Escherichia coli* ATCC 25922	27.12 MHz, 6 kW, 55 °C, 1.5 min	5	[76]
Eggshell	*Escherichia coli* ATCC 35218	27.12 MHz, 3.5 min, 35 °C and hot water (56.7 °C) for 20 min	6.5	[77]
Chunky peanut butter cracker sandwiches	*Escherichia coli* O157:H7	27.12 MHz, 9 kW, 90 s	5.3	[78]
Dried red pepper	*Escherichia coli* O157:H7	27.12 MHz, 9 kW, 50 s	>5	[79]
Black pepper kernels	*Escherichia coli* O157:H7	27.12 MHz, 12 kW, 90 °C, 7 min	>6	[72]
Ground beef	*Escherichia coli* (non-pathogenic cocktail)	27.12 MHz, 6 kW, 55 °C	5	[80]
Infant formula	*Cronobacter sakazakii*	27.12 MHz, 6 kW, 116.5 min, 70 °C (Dry heat)	5	[81]
Salmon caviar	*Listeria innocua*	27.12 MHz, 6 kW, 65 °C, 500 IU/mL nisin	>7	[82]
**Fungi Inactivation**				
**Product**	**Microorganism**	**Processing Conditions**	**Log Reduction**	**Reference**
Peanut kernels	*Aspergillus flavus*	27.12 MHz, 6 kW, and hot air (65 °C—9 min, 0.735 a_w_/70 °C—15 min, 0.876 a_w_)	3.0 and 3.4, respectively	[60]
Wheat seeds	*Aspergillus flavus*	27.12 MHz, 12 kW, and hot air (65 °C—10 min)	2 (when moisture content of seeds was 12%), 3 (when moisture content of seeds was 15%)	[83]
Corn seeds	*Aspergillus flavus*	27.12 MHz, 12 kW, and hot air (65 °C—10 min)	3 (when moisture content of seeds was 12%), 4 (when moisture content of seeds was 15%)	[83]
Corn grains	*Aspergillus parasiticus*	27.12 MHz, 6 kW, and hot air (70 °C—12 min)	5–6	[84]
Enriched white bread	*Penicillium citrinum*	27.12 MHz, 6 kW, and hot air (58 °C—5 min)	4	[85]
Chestnuts	*Penicillium crustosum*	27.12 MHz, 6 kW, and hot air (60 °C)	4	[86]

* Listed by microorganism.

Awuah et al. [87] tested RF to inactivate surrogate microorganisms in milk. The experiment involved strains of *L. innocua* and *E. coli* K12 inoculated in pasteurized whole milk. The treatment was conducted at 27.12 MHz, 2 kW, 65 °C, and 55 s. After processing the milk inside a piece of tubular equipment with a laminar flow, the inactivation was about 5 and 7 log-reduction for *L. innocua* and *E. coli,* respectively.

Eggs have been tested in different presentations using RF. *Salmonella* spp. is the leading pathogen of concern in this product [52]. In-shell eggs, liquid whole egg, egg white powder, liquid egg yolk, and liquid egg white are some examples evaluated under dielectric heating [51,52,68,88]. Table 4 shows some examples of the successful inactivation of microorganisms with RF in different egg products. Geveke et al. [52,77] were able to pasteurize shell eggs using radio frequency, inactivating *S.* Typhimurium and *E. coli* without changes in egg quality. This research group improved the process to reduce the processing time further. The combination of RF plus hot water immersion and RF plus hot water spraying decreased processing time to 19.5 and 24.5 min, ensuring the five log-reduction of the *Salmonella* strain. The processes did not affect the quality and functionality of the egg [50]. 

In Table 4, there is a list of some examples in which RF has been effective in the inactivation of fungi. *Aspergillus* spp. and *Penicillium* spp. are some of the most studied microorganisms. Together with *Fusarium* and *Alternaria*, these species are responsible for forming mycotoxins. These secondary metabolites can be present in food and represent a high risk for human health [89]. Some products with a short shelf life because of fungi spores can be treated with RF without altering the quality attributes but decreasing the spore count. The inactivation of *Aspergillus* spp. and *Penicillium* spp. presented in Table 4 shows that the longest processing time was 15 min. *Monilinia* spp. is a fungi specie that causes significant crop losses, such as peaches and nectarines, and it was studied under RF. Brown rot is a postharvest disease caused by *Monilinia laxa* Honey and *M. fructicola* Honey. Sisquella et al. [90] studied the use of RF (27.12 MHz, 15 kW, 40 °C, 4.5 min) in peaches and nectarines inoculated with *M. fruticola.* After the treatment, the brown rot disease was reduced to less than 10%. 

## 5. Disinfestation

Pest control is essential in the production chain of nuts, cereals, grains, legumes, and seeds. Insects are responsible for damaging these products because of web forming and direct feeding. The most common pests are the codling moth (*Cydia pomonella*), navel orangeworm (*Amyelois transitella*), Indianmeal moth (*Plodia interpunctella*), and red flour beetle (*Tribolium castaneum*) [91,92]. 

Chemicals to control food pests have been used during postharvest activities because they are cheap, fast, and accessible. Some of these chemicals, such as phosphine, are highly toxic to humans, representing a serious health risk, even if found in small food traces. Releasing some of these compounds into the atmosphere after being used for pest disinfestation also has adverse environmental effects. Other food practices to control insect pests include heat, which usually involves a long processing time.

RF has been reported as a successful technology inactivating insects as a postharvest intervention in food products. The first reports of the technology to control pests in food date almost 90 years ago [93]. In the last 20 years, much work has been conducted in this area with successful results and fundamental advances in equipment development. 

The RF energy inactivates insects because of thermal damage to the insect structure’s carbohydrates, proteins, DNA, RNA, and lipids [8]. This technology has been successfully tested for the disinfestation of fresh fruits like nectarine, peach, plum, cherries, apples, oranges, and persimmons; grains such as rice and wheat; legumes such as lentils, black-eyed peas, mung beans, soybeans; dried fruits and nuts such as chestnuts, walnuts, almonds, raisins, dates, apricots, figs, and prunes [8,9].

The use of RF offers a green alternative to eliminating insects in foods. The technology is contactless, chemical, and residue-free and an efficient option in disinfestation. Several reports about the use of RF in insect control are available, showing the elimination of codling moths in cherries and apples, Mexican fruit flies in persimmons, rice weevil *(Sitophilus oryzae*) in milled rice, orangeworm (*Amyelois transitella*) in almonds, among others [94,95]. In Table 5, there are additional examples of insect disinfestation in food products; in all cases, the disinfestation was complete (100%).

As a technology for pest control, the main highlight of RF is the feasibility of using selective or differential heating [95]. As previously mentioned, RF uses dielectric heating to increase the temperature of the product. The two main mechanisms involved in this heating are ionic polarization and dipole rotation. However, selective heating increases the insects’ temperature until they reach a fatal value but maintains moderate heating of the host material or food. This fact considerably reduces any thermal degradation effect on the food quality [93]. 

Wang et al. [100] studied the use of RF to disinfest in-shell walnuts. The main pests found in this product are the codling moth, Indianmeal moth, and navel orangeworm. The RF processing of in-shell walnuts was a short treatment of only 5 min (6 kW, 27.12 MHz, 55 °C) that was able to kill 100% of the most heat-resistant pest, the navel orangeworm. This study also included the analysis of rancidity, sensory attributes, and shell characteristics. There were no changes between the control sample and the RF-processed samples. This is a clear example of how RF can inactivate the pests but protect and preserve the quality of the product because of selective heating. RF also promotes more uniform heating for in-shell products like walnuts. During conventional heating processes, the shell produces an isolation effect on the kernel extending the processing time. In the case of RF, the heating is generated in the shell and the kernel simultaneously, reducing the processing time and allowing a more uniform treatment. 

## 6. Food Quality

As previously mentioned, RF surged as an alternative to conventional thermal treatment to reduce the undesirable effects on quality. Most studies conducted with this novel technology included a detailed examination of quality attributes after processing. The quality of the product is not impacted during RF because of the different approaches used during processing, such as the movement of the development, change in electrodes, and change in packaging materials, among others. Some of these are summarized in this section. 

Dragon fruit or pitaya was treated with RF, and several quality attributes were studied after processing and during the shelf life. Shen et al.’s [101] primary goal was to identify the best treatment regarding the gaps between the electrode plates of the system that had the minimum impact on the quality of dragon fruit slices. The treatment (6 kW, 27.12 MHz, 10 min, 70 °C) delayed some biochemical reactions and microbial growth for 2–3 days during storage. This product has a high moisture content (82.63%) which increases the rate of enzymatic activity and microbial growth. Some changes were observed in color. However, as in many other novel technologies, some bioactive compounds were increased after the treatment. In the case of dragon fruit, the total phenolics were increased after RF. The main reason could be the new structure of the product after processing that allows for a more straightforward quantification of the phenolics, releasing these compounds from intercellular structures. A similar fact was observed when kiwi puree was treated with RF treatments (27.12 MHz, 10 kW). The process delayed microbial growth and improved the quality of the product. A higher concentration of vitamin C, total phenolic compounds, and antioxidant capacity was found after RF processing. The panelists preferred the RF-treated product rather than the thermal process in terms of sensory quality. The color was also better during seven weeks of storage for the RF-treated food [102]. 

A study is mentioned in Table 4 about the pasteurization of peanut butter sandwiches, a potentially challenging RTE product, because of its complex, heterogeneous nature. In this research, the inactivation of pathogens was conducted in chunky and creamy peanut butter cracker sandwiches using RF. However, there was also a comprehensive evaluation of the product after processing. The color was evaluated in peanut butter and the cracker surface after the longest processing time (27.12 MHz, 9 kW, 90 s), and no changes were detected using analytical techniques. Furthermore, a sensory panel was conducted with the control and processed samples to evaluate any possible flavor, texture, and overall acceptability changes. The results showed no significant differences between the control and RF-processed samples [78]. 

Liao et al. [103] studied RF as a processing aid to stabilize wheat germ. This product is a rich ingredient in proteins, carbohydrates, fatty acids, and tocopherols that needs to be stabilized to extend the shelf life. The main problems during storage are oxidative and hydrolytic enzymatic reactions. During the RF processing (12 kW, 27.12 MHz), lipase, peroxidase, polyphenol oxidase, and lipoxygenase were inactivated. The extracted oils showed peroxide and acid values stable during the first three days of storage (25 °C), and no changes were detected in the α-tocopherol, amino acid, or fatty acid content. The process was better when the wheat germ was processed with an initial moisture content of about 15%. 

A similar study was conducted by Xu et al. [74] on buckwheat. The main goal was to inactivate the natural flora in this product using RF (27.12 MHz, 6 kW). The tested temperature was 70 to 90 °C, and the processing time did not exceed 20 min. The natural flora composed of mesophiles, *Enterobacteriaceae*, yeasts, and molds showed a high initial value (>6 logs). The RF treatment was able to inactivate about three log reductions of natural microflora. Buckwheat was also inoculated with pathogens (*S.* Typhimurium, *E. coli*, *C. sakazakii, B. cereus*), and the treatment also inactivated about four log-reduction of pathogens. The quality studies showed that color was not affected after the RF treatment, using the strongest temperature conditions, and nutrient loss was not detected in the product compared to control samples. 

Ling et al. [104] improved the efficiency of RF equipment to disinfest pistachios. This research team was able to inactivate 100% of the Indianmeal moth in the product. They used an RF system (27.12 MHz, 6 kW) that could kill all the insects in just a few minutes. For the in-shell pistachios in a 1.8 kg bag, the disinfestation was achieved in 5.6 min. Meanwhile, for the pistachios, shelled in a 2 kg bag, the time was only 5.5 min. These processes were more efficient than conventional hot air processing, which took 82 and 117 min, respectively. The authors evaluated the stability of the product during the storage, and there were no significant differences between control and RF-treated samples. The evaluated parameters were weight loss, peroxide values, fatty acid composition, and kernel color. In a similar study with the same pest but in-shell walnuts, RF processing (27.12 MHz, 25 kW, 52 °C) could fully disinfest the product after 5 min. Two kinds of products were used, the unwashed and the air-dried walnuts packaged in a polyethylene container. Walnuts were studied over storage for 20 days at 35 °C, and no changes were observed in kernel color, peroxide, or fatty acid values. This storage under temperature abuse conditions is equivalent to 2 years of commercial storage at 4 °C [100]. 

RF can also be used to change the functionality of some biomolecules with a specific goal. These changes can be later used for novel product development or to improve the functionality of some products during food processing. A detailed study on RF on soy protein isolate (SPI) dispersion observed some changes in the protein structure after processing. The treatment applied at 6 kW, 27.12 MHz, and 90 °C changed the hydrophobicity of the SPI, reducing its hydration. Studies showed that the tertiary and secondary structures were changed, but the primary structure remained intact. The SPI was self-reassembled from the coil to a β-sheet structure, modifying its functionality [105]. 

In general terms, RF is a technology with several uses in the food industry. The advantages of this novel thermal technology are numerous compared to the conventional thermal process, as has been mentioned in the text. Other emerging technologies such as microwave, ohmic heating, or induction heating are also potential technologies to replace the conventional thermal process in the future. Although the description of these technologies is out of the scope of this manuscript, it is worth mentioning that each technology has specific uses for certain food products. For example, RF and microwave follow a similar heating mechanism at different frequencies, but the penetration depth in the product is a decision point to choose between technologies. 

## 7. Conclusions

RF has been widely studied in recent decades, offering an alternative to conventional thermal processing. Knowledge about the dielectric properties of very diverse products has been published. Without a doubt, as of today, the main hurdle in using this technology is the nonuniformity of the product. Poor uniform heating can reduce product quality and create a safety risk from the microbial point of view. Much research is now focused on improving processing equipment and process optimization to address this hurdle. The constant work together with packaging scientists is also filling those gaps that will allow processing the food using RF in pre-packed containers. Like other novel technologies, each food represents a diverse and complex material to study and characterize because of its shape, size, composition, and structure. Numerical simulation has become a powerful tool in studying and developing processes and equipment. The regulatory aspects of RF related to pasteurization need to be carefully reviewed to be approved as an official technology for this sole purpose. RF is a green technology for pasteurization and disinfestation that can achieve food safety targets without changing product quality. 

## Figures and Tables

**Table 1:** Dielectric properties of fruits and vegetables at selected temperatures in the radio frequency range (27.12 and 40.68 MHz).

Product	Temperature (°C)	Moisture Content (%)	Dielectric Constant (*ε*′)		Loss Factor (*ε*″)		Penetration Depth (*m*)		Reference
			27.12 MHz	40.68 MHz	27.12 MHz	40.68 MHz	27.12 MHz	40.68 MHz	
Golden Delicious apple (pulp)	20	-	63.58	-	67.88	-	-	-	[11]
Red Delicious apple (pulp)	20	-	62.38	-	67.37	-	-	-	[11]
Red Delicious apple	20 40 60	87	74.6 70.6 66.8	74.7 70.8 66.8	92.0 130.7 178.6	61.1 87.5 119.9	0.189	-	[12]
Apple juice	25 55 85	-	81.9 74.7 68.9	79.9 71.5 64.4	135.5 224.3 314.8	88.7 146.5 205.4	-	-	[13]
Apricots (dried)	20 40 60	24.6	33.9 37.4 40.8	32.3 35.7 38.9	11.8 19.9 37.4	10.6 16.2 28.9	88.8 × 10^−2^ 56.3 × 10^−2^ 32.8 × 10^−2^	64.9 × 10^−2^ 45.0 × 10^−2^ 27.4 × 10^−2^	[14]
Avocado (pulp)	20	-	146.73	-	574.71	-	-	-	[11]
Avocado	20 40 60	-	115.7 131.6 140.5	92.7 100.0 105.4	699.6 951.6 1422.0	477.2 648.6 965.1	5.1 × 10^−2^	-	[15]
Cherimoya	20 40 60	-	71.5 68.4 70.0	-	68.6 64.5 65.4	-	9.4 × 10^−2^	-	[15]
Dates	20 40 60	19.7	27.2 31.0 35.0	25.5 28.9 32.9	10.1 15.0 26.9	9.0 12.2 20.0	93.0 × 10^−2^ 67.5 × 10^−2^ 41.4 × 10^−2^	68.2 × 10^−2^ 53.9 × 10^−2^ 35.7 × 10^−2^	[14]
Figs (dried)	20 40 60	27.3	37.7 42.3 46.5	35.7 40.1 44.2	14.4 23.8 42.2	13.1 19.2 32.7	76.7 × 10^−2^ 50.0 × 10^−2^ 31.0 × 10^−2^	55.5 × 10^−2^ 40.5 × 10^−2^ 25.7 × 10^−2^	[14]
Grape juice	25 55 85		81.3 74.6 68.8	79.1 70.9 63.5	209.1 339.8 507.2	136.7 221.6 330.7	-	-	[13]
Grapefruit (pulp)	20	-	99.42	-	245.7	-	-	-	[11]
Kiwi slices (airdried)	20 80 20 80	59.5 70	81.49 90.68 97.53 96.74	74.12 82.98 88.62 86.06	332.14 777.66 407.45 808.19	235.17 546.03 284.32 576.96	7.71 × 10^−2^ 4.73 × 10^−2^ 6.94 × 10^−2^ 4.64 × 10^−2^	6.42 × 10^−2^ 3.89 × 10^−2^ 5.83 × 10^−2^ 3.78 × 10^−2^	[16]
Kiwi slices (osmotic dehydrated)	20 80 20 80	60.5 70.2	73.15 82.00 91.28 89.71	67.46 75.91 82.48 80.76	291.92 703.63 371.19 757.67	201.50 494.31 258.78 545.84	8.25 × 10^−2^ 4.97 × 10^−2^ 7.30 × 10^−2^ 4.80 × 10^−2^	7.01 × 10^−2^ 4.10 × 10^−2^ 6.14 × 10^−2^ 3.89 × 10^−2^	[16]
Longan	20 40 60	-	75.2 71.6 67.5	73.8 69.5 65.0	230.1 326.4 431.4	156.5 221.9 293.3	9.7 × 10^−2^	-	[15]
Orange juice	25 55 85	-	83.5 76.4 65.4	81.1 72.6 60.3	222.1 372.4 522.5	144.9 242.5 340.0	-	-	[13]
Orange, navel (pulp)	20	-	84.57	-	222.48	-	-	-	[11]
Orange, Valencia (pulp)	20	-	85.29	-	240.09	-	-	-	[11]
Passion fruit	20 40 60	-	82.7 88.1 96.6	73.5 74.7 77.7	264.1 373.6 523.9	179.7 254.1 356.3	9.0 × 10^−2^	-	[15]
Peach (pulp)	20	-	90.09	-	269.5	-	-	-	[11]
Pear juice	25 55 85	-	80.9 71.2 61.9	79.7 69.4 59.8	182.2 300.5 435.8	119.1 196.0 284.2	-	-	[13]
Persimmon	20 40 60	-	76.0 75.5 69.1	73.6 72.2 64.9	258.6 369.9 470.8	176.0 251.3 319.4	10.5 × 10^−2^	-	[15]
Pineapple juice	25 55 85	-	84.9 75.1 65.5	81.2 69.4 58.1	276.8 436.2 586.2	180.7 284.5 382.3	-	-	[13]
Potatoes	20 40 60	83.3 79.8 73.3	48.7 57.2 118.2	-	293.5 623.4 693.0	-	-	-	[17]
Potatoes, mashed (0.8% NaCl)	20 60 120	84.7	88.6 89.9 102.8	82.4 79.5 81.6	297.5 541.0 1153.8	203.6 367.9 782.4	84.0 × 10^−3^ 58.4 × 10^−3^ 38.5 × 10^−3^	72.1 × 10^−3^ 49.0 × 10^−3^ 31.8 × 10^−3^	[18]
Potatoes, mashed (1.8% NaCl)	20 60 120	85.9	78.2 79.9 112.2	71.4 68.6 84.3	713.3 1306.7 3152.2	480.5 878.2 2104.4	49.5 × 10^−3^ 35.7 × 10^−3^ 22.8 × 10^−3^	41.5 × 10^−3^ 29.6 × 10^−3^ 18.8 × 10^−3^	[18]
Prunes (dried)	20 40 60	30.2	40.6 44.4 48.9	38.7 42.7 47.2	17.2 25.4 47.8	15.7 20.6 38.4	66.9 × 10^−2^ 48.1 × 10^−2^ 28.4 × 10^−2^	48.3 × 10^−2^ 38.9 × 10^−2^ 22.9 × 10^−2^	[14]
Raisins	20 40 60	15	21.9 28 33.8	20.2 26.1 31.9	8.1 9.8 11.4	7.4 9.0 10.6	103.7 × 10^−2^ 96.9 × 10^−2^ 91.3 × 10^−2^	74.0 × 10^−2^ 68.7 × 10^−2^ 64.4 × 10^−2^	[14]
White sapote	20 40 60	-	76.0 75.5 69.1	73.6 72.2 64.9	258.6 369.9 470.8	176.0 251.3 319.4	9.0 × 10^−2^	-	[15]

**Table 2:** Dielectric properties of animal food products at selected temperatures in the radio frequency range (27.12 and 40.68 MHz).

Product	Temperature (°C)	Moisture Content (%)	Dielectric Constant (*ε*′)		Loss Factor (*ε*″)		Penetration Depth (*m*)		Reference
			27.12 MHz	40.69 MHz	27.12 MHz	40.68 MHz	27.12 MHz	40.68 MHz	
Meat									
Beef meatball	20 60 100	66.9	68.8 79.1 95.8	56.6 69.1 79.0	474.4 922.0 1557.5	323.9 625.4 1054.1	61.7 × 10^−3^ 43.0 × 10^−3^ 32.7 × 10^−3^	51.1 × 10^−3^ 35.6 × 10^−3^ 27.0 × 10^−3^	[19]
Beef (lean)	Heating	71.5	70.5	-	418.7	-	0.132	-	[20]
Lamb (lean)	Heating	73	77.9	-	387.2	-	0.140	-	[20]
Pork (lean)	Heating	73.9	69.6	-	392.0	-	0.137	-	[20]
Pork (fat)	Heating	19.0	12.5	-	13.1	-	1.054	-	[20]
Lean beef (11.8% fat content)	5 10	67.8	74 72	-	290 310	-	0.100 0.100	-	[21]
Beef (50:50, 36.1% fat content)	5 10	48.2	42 40	-	110 120	-	0.260 0.220	-	[21]
Fatty beef (65.7% fat content)	5 10	26.3	19 18	-	20 18	-	0.580 0.620	-	[21]
Poultry									
Chicken breast	20 60 100	75.1	91.64 109.18 126.03	83.50 94.59 106.28	332.33 567.21 618.96	227.34 388.57 427.76	78.55 × 10^−3^ 57.74 × 10^−3^ 55.57 × 10^−3^	66.96 × 10^−3^ 48.27 × 10^−3^ 46.12 × 10^−3^	[22]
Chicken (lean)	Heating	73.6	75.0	-	480.8	-	0.123	-	[20]
Turkey (lean)	Heating	74.5	73.5	-	458.4	-	0.126	-	[20]
Eggs									
Liquid egg whites	20 60 100	85	84.6 81.3 118.1	76.6 68.0 88.9	427.0 646.4 1242.3	256.4 427.7 784.3	-	-	[23]
Pre-cooked egg whites	20 60 100	-	89.3 92.5 106.8	81.9 82.3 88.2	411.8 732.1 1145.0	256.3 460.0 698.9	-	-	[23]
Liquid whole egg	20 60 100	73.7	76.3 77.4 96.9	68.8 66.0 77.5	335.9 612.0 956.4	208.4 377.1 589.8	-	-	[23]
Pre-cooked whole eggs	20 60 100	-	79.6 85.2 94.2	71.0 72.1 75.4	336.8 594.1 874.1	209.5 367.4 539.5	-	-	[23]
Egg albumen	24	87.8	89	81	507	362	-	-	[24]
Egg yolk	24	52.1	56	51	200	145	-	-	[24]
Dairy									
Mozzarella cheese	20 60 100	59.1	55.6 74.6 82.2	49.4 63.0 66.1	358.5 853.6 1266.9	245.5 579.9 858.9	71.3 × 10^−3^ 44.7 × 10^−3^ 36.3 × 10^−3^	59.5 × 10^−3^ 37.0 × 10^−3^ 29.9 × 10^−3^	[19]
Raw milk	20 60 120	88.20	90.4 93.3 109.3	83.3 80.0 84.5	299.8 562.6 979.8	203.6 379.8 661.9	0.098 0.068 0.050	0.076 0.048 0.038	[25]
Skimmed milk	20 60 120	90.44	89.7 91.1 102.0	84.5 80.6 83.3	310.0 590.6 1020.2	209.4 399.0 690.1	0.096 0.065 0.040	0.072 0.048 0.036	[25]
Whole milk powder	20 50 90	1.8	1.51 2.09 4.46	-	0.007 0.100 2.511	-	-	-	[26]
Non-fat milk powder	20 50 90	4.8	1.12 2.00 3.16	-	0.004 0.100 1.580	-	-	-	[26]
Concentrated non-fat milk (35%)	20 60 120	65.45	99.1 119.6 152.9	87.5 99.0 117.6	536.8 1121.6 1953.5	365.6 760.0 1319.2	0.069 0.048 0.038	0.052 0.032 0.028	[25]
Concentrated milk 70% 85% 100%	22	-	76.4 76.3 75.8	-	233.8 266.4 282.1	-	0.142 - 0.130	-	[27]
Fish and seafood									
Salmon	20 60 100	75.7	77.61 96.84 116.37	-	462.66 809.98 1185.56	-	63.10 × 10^−3^ 46.61 × 10^−3^ 38.11 × 10^−3^	-	[22]
Salted (2.3%) salmon caviar	20 50 80	-	129.8 121.5 182.0	-	1349.4 1501.1 2614.5	-	3.7 × 10^−2^ 3.4 × 10^−2^ 2.6 × 10^−2^	-	[28]
Unsalted (0.8%) salmon caviar	20 50 80	-	70.7 46.4 59.6	-	470.8 375.9 642.7	-	6.3 × 10^−2^ 7.2 × 10^−2^ 5.5 × 10^−2^	-	[28]
Salted (3.3%) sturgeon caviar	20 50 80	-	81.5 111.5 202.8	-	1004.0 1769.5 2873.3	-	4.2 × 10^−2^ 3.1 × 10^−2^ 2.5 × 10^−2^	-	[28]
Unsalted (0.2%) sturgeon caviar	20 50 80	-	61.0 77.4 92.5	-	105.5 210.8 352.2	-	16.0 × 10^−2^ 11.0 × 10^−2^ 7.80 × 10^−2^	-	[28]
Trout	20 60 100	72.8	83.64 103.55 100.34	-	343.83 645.04 806.62	-	76.02 × 10^−3^ 53.30 × 10^−3^ 46.82 × 10^−3^	-	[22]

**Table 3:** Dielectric properties of miscellaneous food products at selected temperatures in the radio frequency range (13.56 and 27.12 MHz).

Product	Temperature (°C)	Moisture Content (%)	Dielectric Constant (*ε*′)		Loss Factor (*ε*″)		Penetration Depth (*m*)		Reference
			13.56 MHz	27.12 MHz	13.56 MHz	27.12 MHz	13.56 MHz	27.12 MHz	
Flour, starch, bread									
Chestnut flour (compressed)	20 40 60	45.3	-	31.2 38.8 57.7	-	45.9 77.9 158.1	-	0.280 0.190 0.130	[29]
Chickpea flour	20 50 90	7.9	-	2.99 3.44 11.20	-	0.16 0.19 4.27	-	17.92 - 13.6	[30,31]
Chickpea flour	20 50 90	20.9	-	4.50 11.43 71.59	-	0.81 7.85 248.25	-	44.9 - 0.088	[29,30]
Green pea flour	20 60 90	21.6	-	7 28 85	-	1.4 20 180	-	3.61 0.52 0.146	[31]
Lentil flour	20 60 90	21.5	-	5.5 30 100	-	1 25 200	-	4.31 0.42 0.12	[31]
Potato starch	-	-	5.72	5.56	0.14	0.1	1.08	0.55	[32]
Soybean flour	20 60 90	19.9	-	6 20 70	-	1.5 20 200	-	2.70 0.39 0.09	[31]
Tapioca flour	-	-	4.07	3.91	0.13	0.09	1.31	0.67	[32]
Wheat flour	25 55 85	12.56	-	5.61 6.58 23.50	-	5.42 6.25 20.47	-	-	[33]
Wheat flour with 10% bran content	25 55 85	12.56	-	0.54 0.62 9.74	-	0.54 0.60 9.39	-	-	[33]
Wheat germ	25 55 85	7.05	2.78 3.46 5.39	2.64 3.37 5.14	0.30 0.43 0.68	0.29 0.39 0.62	-	-	[34]
White bread	25 55 85	34.6	2.76 3.37 4.22	2.35 2.80 3.45	4.56 10.70 26.55	2.32 5.09 11.98	2.105 1.206 0.710	0.1283 0.721 0.417	[35]
Nuts									
Almonds (ground shells)	20 50 90	6	-	2.07 2.18 4.42	-	0.10 0.12 1.35	-	24.82 - 2.78	[36]
Almonds (ground shells)	20 50 90	36	-	8.96 13.41 26.91	-	12.43 38.48 92.30	-	0.496 - 0.150	[36]
Almonds (ground shells)	25 60 100	12	-	9.3 11.1 13.8	-	5.7 11.0 26.0	-	98.9 × 10^−2^ 58.6 × 10^−2^ 31.5 × 10^−2^	[36]
Macadamia nuts	25 60 100	24	-	20.6 22.9 29.2	-	47.5 81.0 173.5	-	22.3 × 10^−2^ 15.9 × 10^−2^ 10.3 × 10^−2^	[37]
Peanut kernels	25 85	10	-	6.2 7	-	0.8 1.15	-	5.24 3.79	[38]
Peanut kernels	25 85	30	-	25 35	-	50 125	-	0.22 0.12	[38]
Pecan kernels (no salt)	25 65	15	-	8.97 20.01	-	3.35 15.19	-	6.07 1.29	[39]
Pecan kernels (light salt)	25 65	15	-	11.46 23.71	-	8.58 27.02	-	69.95 29.54	[39]
Pecan kernels (medium salt)	25 65	15	-	13.97 27.43	-	14.96 34.83	-	51.69 22.79	[39]
Pecan kernels (heavy salt)	25 65	15	-	15.48 29.37	-	24.26 47.96	-	42.69 13.76	[39]
Pistachio (non-salted)	24	3.5	-	10.37	-	5.33	-	0.53	[40]
Pistachio (100 mg sodium/serving)	24	4.08	-	15.34	-	15.83	-	0.31	[40]
Pistachio (330 mg sodium/serving)	24	3.75	-	23.78	-	42.83	-	0.19	[40]
Pistachio kernels: non-salted	25 85	15	-	11.85 17.74	-	5.99 23.09	-	104.27 × 10^−2^ 36.93 × 10^−2^	[41]
Pistachio kernels: light salted	25 85	15	-	15.73 24.65	-	15.65 63.36	-	49.06 × 10^−2^ 18.92 × 10^−2^	[41]
Pistachio kernels: medium salted	25 85	15	-	21.41 32.55	-	24.92 82.66	-	36.85 × 10^−2^ 16.60 × 10^−2^	[41]
Pistachio kernels: strong salted	25 85	15	-	22.15 31.42	-	44.82 133.21	-	23.60 × 10^−2^ 12.13 × 10^−2^	[41]
Species									
Chili powder	-	-	7.22	6.85	0.61	0.39	0.95	0.49	[32]
Cumin	-	9.6	2.1	2.0	-	-	-	-	[42]
Curry	-	8.3	2.1	2.0	0.01	0	-	-	[42]
Garlic	-	3.1	1.8	1.7	-	-	-	-	[42]
Onion powder	-	-	2.22	2.18	-	-	-	-	[32]
Paprika	-	12.3	3.6	3.4	0.20	0.18	-	-	[42]
Black pepper	-	10.4	3.0	2.8	0.10	0.05	-	-	[42]
Red pepper	-	11.5	2.8	2.2	0.1	0.07	-	-	[42]
Red pepper powder	25 35 55 85	17.6	-	6.63 7.55 9.83 23.28	-	2.00 2.80 5.76 50.69	-	3.00	[43]
White pepper	-	10.2	3.6	3.4	0.18	0.12	-	-	[42]
Turmeric	23	9.5	2.8	2.6	0.12	0.09	-	-	[42]
Others									
Black-eyed pea	20 40 60	16.8	-	3.64 4.18 6.67	-	0.40 0.60 1.67	-	8.42 6.03 2.76	[44]
Broccoli powder	20 40 60 80	9.1	4.66 5.78 8.75 12.48	4.21 5.58 8.22 11.35	0.17 0.28 0.81 2.1	0.12 0.19 0.54 1.34	12.48 7.95 3.43 1.88	8.86 5.17 2.48 1.27	[32]
Edible fungi powder (*Pleurotus eryngii*)	25 55 85	12.0	4 7 26	-	1 3 36	-	4.30 2.42 0.29	-	[45]
Edible fungi powder (*Pleurotus eryngii*)	25 55 85	21.2	8 30 70	-	0.001 50 460	-	2.19 0.24 0.072	-	[45]
Edible fungi powder (*Pleurotus eryngii*)	25 55 85	30.9	20 60 142	-	10 180 740	-	0.430 0.110 0.051	-	[45]
Honey (Jujube)	25	17.5	-	30.70	-	8.06	-	-	[46]
Honey (Yellow-locust)	25	18.1	-	32.45	-	8.74	-	-	[46]
Honey (Milk vetch)	25	17.1	-	30.98	-	8.27	-	-	[46]
Lasagna sauce	20 60 100	92.0	-	86.2 77.7 85.6	-	1045.3 1862.1 3043.7	-	40.3 × 10^−3^ 29.6 × 10^−3^ 23.0 × 10^−3^	[19]
Lasagna noodles	20 60 100	60.7	-	92.5 85.2 85.1	-	516.8 943.3 1496.6	-	60.1 × 10^−3^ 42.6 × 10^−3^ 33.2 × 10^−3^	[19]
Mung bean	20 40 60	14.4	-	4.21 4.50 6.07	-	0.43 0.54 1.11	-	8.44 6.93 3.93	[44]

**Table 5:** Examples of disinfestation in food products using radio frequency.

Product	Organism	Processing conditions	Disinfestation Level	Reference
Rapeseeds	Red flour beetle (*Tribolium castaneum*)	27.12 MHz, 1.5 kW, 80 °C	100%	[96]
Milled, rough, and brown rice	Rice weevil (*Sitophilus oryzae*)	27.12 MHz, 12 kW, 50 °C, 5 min	100%	[97]
Rough, brown, and milled rice	*Rhyzopertha dominica* (Fabricius)	27.12 MHz, 15 kW, 54 °C, 11 min	100%	[98]
Walnuts	Rice moth larvae (*Corcyra cephalonica* L.)	6.78 MHz, 13.56 MHz, 27.12 MHz, 40.68 MHz, 2 kW, 70–76 °C, 20 min	100%	[99]

## Data Availability

Data is contained within the article.

## Statement

The mention of trade names or commercial products in this article is solely for the purpose of providing specific information. It does not imply a recommendation or endorsement by the U.S. Department of Agriculture. USDA is an equal opportunity provider and employer.
